# Cathepsin X Activity Does Not Affect NK-Target Cell Synapse but Is Rather Distributed to Cytotoxic Granules

**DOI:** 10.3390/ijms222413495

**Published:** 2021-12-16

**Authors:** Tanja Jakoš, Mateja Prunk, Anja Pišlar, Janko Kos

**Affiliations:** 1 Department of Pharmaceutical Biology, Faculty of Pharmacy, University of Ljubljana, 1000 Ljubljana, Slovenia; tanja.jakos@gmail.com (T.J.); anja.pislar@ffa.uni-lj.si (A.P.); 2 Department of Biotechnology, Jožef Stefan Institute, 1000 Ljubljana, Slovenia; mateja.prunk@ijs.si

**Keywords:** cytotoxic cells, cathepsin X, NK-92, immunological synapse, LFA-1

## Abstract

Cathepsin X is a lysosomal peptidase that is involved in tumour progression and represents a potential target for therapeutic interventions. In addition, it regulates important functions of immune cells and is implicated in the modulation of tumour cell–immune cell crosstalk. Selective cathepsin X inhibitors have been proposed as prospective antitumour agents to prevent cancer progression; however, their impact on the antitumour immune response has been overlooked. Previous studies indicate that the migration and adhesion of T cells and dendritic cells are affected by diminished cathepsin X activity. Meanwhile, the influence of cathepsin X inhibition on natural killer (NK) cell function has not yet been explored. Here, we examined the localization patterns of cathepsin X and the role of its inhibitors on the cytotoxicity of cell line NK-92, which is used for adoptive cellular immunotherapy in cancer patients. NK-92 cells depend on lymphocyte function-associated antigen 1 (LFA-1) to form stable immunoconjugates with target cells, providing, in this way, optimal cytotoxicity. Since LFA-1 is a substrate for cathepsin X activity in other types of cells, we hypothesized that cathepsin X could disturb the formation of NK-92 immunoconjugates. Thus, we employed cathepsin X reversible and irreversible inhibitors and evaluated their effects on the NK-92 cell interactions with target cells and on the NK-92 cell cytotoxicity. We show that cathepsin X inhibition does not impair stable conjugate formation or the lytic activity of NK-92 cells. Similarly, the conjugate formation between Jurkat T cells and target cells was not affected by cathepsin X activity. Unlike in previous migration and adhesion studies on T cells, in NK-92 cells cathepsin X was not co-localized with LFA-1 at the plasma membrane but was, rather, redistributed to the cytotoxic granules and secreted during degranulation.

## 1. Introduction

Since their discovery in the 1950s, lysosomes have been associated with key cellular homeostatic mechanisms, including, but not limited to, the degradation and recycling of extracellular and intracellular material. Importantly, lysosomes are dynamic organelles, capable of integrating and responding to various signals by sensing cellular energy status, nutrient levels, growth factor signalling, membrane damage and activation of the immune system. Perturbations in lysosomal function are, therefore, reflected in altered signalling pathways that support the development and progression of many diseases, including neurodegeneration, autoimmune disorders and cancer [1,2]. Lysosomes are rich in hydrolases and contain a group of 11 peptidases termed cysteine cathepsins, that are implicated in the regulation of several immune cell functions, from activation of innate immune cells by Toll-like receptor signalling [3,4], cytokine secretion [5,6], phagocytosis [7], to the priming of adaptive immune cells by antigen processing and presentation [8] and by providing optimal cytotoxicity to effector lymphocytes [9]. Cysteine cathepsin expression is upregulated in wide variety of tumours. Numerous genetic and pharmacological studies have observed that inhibition of cathepsin activity could decrease cancer progression, thus establishing cysteine cathepsins as promising therapeutic targets [10,11]. Preclinical data support the use of cathepsin inhibitors to combat cancer, however, few reports indicate that inhibition of cysteine cathepsins would not only affect their tumour-promoting functions, but could also elicit alterations in the antitumour immune response.

Normally, cathepsin X is predominantly expressed in the cells of immune system, especially of myeloid lineage [12,13]. Cathepsin X overexpression was demonstrated in several cancers, including prostate [14], gastric [15], hepatocellular [16], breast [17] and colorectal cancer [18], where it fuels tumour growth by conferring resistance to apoptosis through interaction with RPLP0 [19] and insulin growth factor like 1 receptor [20], by promoting invasion through interaction with integrin receptors ανβ3 and ανβ5 [21,22] and by the cleavage of tumour suppressor protein profilin-1 [23], as well as by enabling the epithelial-to-mesenchymal transition [24]. Recently, it was shown that cathepsin X influences the reciprocal interactions between tumour cells and tumour promoting myeloid-derived suppressor cells (MDSC). Pharmacological tools for counterbalancing excessive proteolytic activity in tumour tissue might therefore have unexpected consequences for the function of immune cells [25]. Furthermore, the leukocyte specific β2 integrin receptors can be sequentially trimmed at C-terminus by cathepsin X, which modulates their association with adaptor proteins (talin, α-actinin) and fine-tunes their affinity for extracellular ligands [26]. Accordingly, cathepsin X inhibition was shown to reduce β2 integrin-dependent adhesion and phagocytosis of macrophages by interaction with macrophage antigen-1 (CD11b/CF18, Mac-1) [27], and to prevent adhesion-dependent maturation of dendritic cells [28]. On the other hand, the overexpression of cathepsin X in T lymphocytes increased their migration and the formation of prolonged extensions [29,30].

During immunological synapse formation, cytotoxic cells use β2 integrin adhesion molecule, lymphocyte function-associated antigen 1 (LFA-1), to anchor to the target cell membrane and to enhance cytotoxic cell activation [31,32]. LFA-1 neutralizing antibodies or inhibitors could block activation signals, thus reducing the adhesion and cytotoxicity of natural killer (NK) cells [33]. It is not yet clear whether cathepsin X could also fine-tune the affinity of LFA-1 during immunological synapse formation and if cathepsin X inhibition could influence the killing potential of cytotoxic cells. This information would be of great importance in underlining the effect of cathepsin X inhibitors on the ability of cytotoxic cells to engage their targets and to assess their potential in chemo- and immunomodulatory cancer therapies.

Genetically engineered NK-92 cells are becoming important tool in cancer immunotherapy, providing off-the-shelf cell preparations for targeted tumour cell elimination [34]. Clonally expanded NK cell lymphoma cells, NK-92, have several advantages over blood preparations of primary NK cells or engineered T cells, and have been demonstrated safe for use in cancer patients [35,36]. Notably, NK cells are superior to cytotoxic T cells in targeting dedifferentiated cancer stem cells [37] and have potent antileukemia activity [38]. In this study, we aimed to elucidate whether the loss of cathepsin X activity, on account of pharmacological inhibition, influences the affinity of LFA-1 in NK-92 cells, and consequently impacts their conjugation with target cells. By using imaging flow cytometry, we investigated the formation of immunological synapses, as well as cytotoxicity in the presence of selective cathepsin X inhibitors. Since the previous studies have revealed the importance of intact cathepsin X activity for regulation of CD4+ T cell migration and adhesion, we also tested the relevance of cathepsin X for immunological synapse stability of CD4+ T cells. Our results did not confirm the involvement of cathepsin X in LFA-1 mediated regulation of immune synapse. However, by investigating cellular compartmentalization of cathepsin X in NK-92 cells, we demonstrate that cathepsin X preferentially colocalizes with perforin during cytotoxic granule release and is, thus, transferred to the target cells.

## 2. Results

### 2.1. Regulation of Cathepsin X Expression and Activity in Activated NK-92 Cells

**Figure 1:**
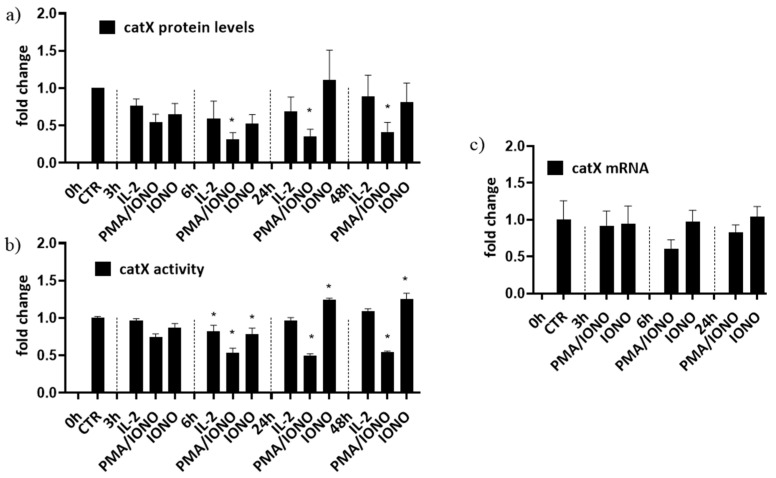
The relative protein levels (**a**), activity (**b**) and mRNA expression (**c**) of mature form of cathepsin X in NK-92 cells. NK-92 cells were either left unstimulated or were stimulated with IL-2, IL-2 plus PMA/IONO or IONO. After indicated time points, cell lysates were prepared to be assayed by Western blot, kinetic assay or by qPCR. The values were normalized to unstimulated cells at time 0 h. The figures represent mean ± SD of three independent biological replicates. (*)—statistically significant difference in comparison with the control.

First, to simulate different activation states of NK cells, NK-92 cells were treated with interleukin 2 (IL-2), with IL-2 plus phorbol myristate acetate/ionomycin (PMA/IONO; which initiate NK-92 cell activation in the absence of target cells) or with IONO alone (which promotes NK cell anergy). Cell lysates were then analysed for the intracellular activity and protein levels of cathepsin X [39]. Only the activation with a combination of PMA/IONO resulted in a significant decrease of cathepsin X activity and protein levels (Figure 1a). Cathepsin X mRNA transcripts, on the other hand, remained unchanged (Figure 1b). Of note, due to significant increase in cathepsin X activity after 24 h treatment with IONO, we also performed cytotoxicity assay on IONO-treated NK-92 cells with or without selective cathepsin X inhibitor Z9, and while there was significant reduction of cytotoxicity due to IONO treatment, there was no difference due to the inhibition of cathepsin X (Supplementary Figure S1).

### 2.2. Cathepsin X Is Secreted upon Stimulation

**Figure 2:**
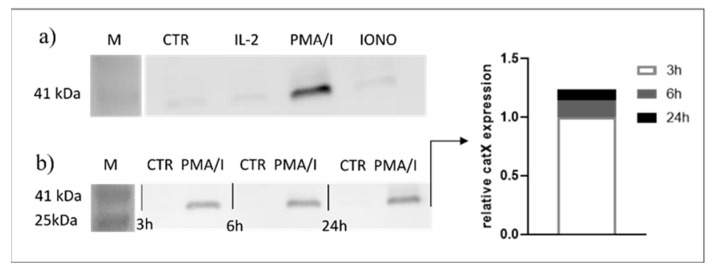
Only activated NK-92 cells secrete cathepsin X. NK-92 cells were seeded in SFM and treated with vehicle (CTR), IL-2, IL-2 plus PMA/IONO or IONO. After the indicated time points, the cell-culture supernatants were collected, concentrated with ultrafiltration and assayed for the presence of cathepsin X by Western blot. Only PMA/IONO-activated NK-92 cells abundantly secreted cathepsin X during 24 h incubation (**a**) and the majority of cathepsin X was secreted in the first 3 h after stimulation (**b**).

**Figure 3:**
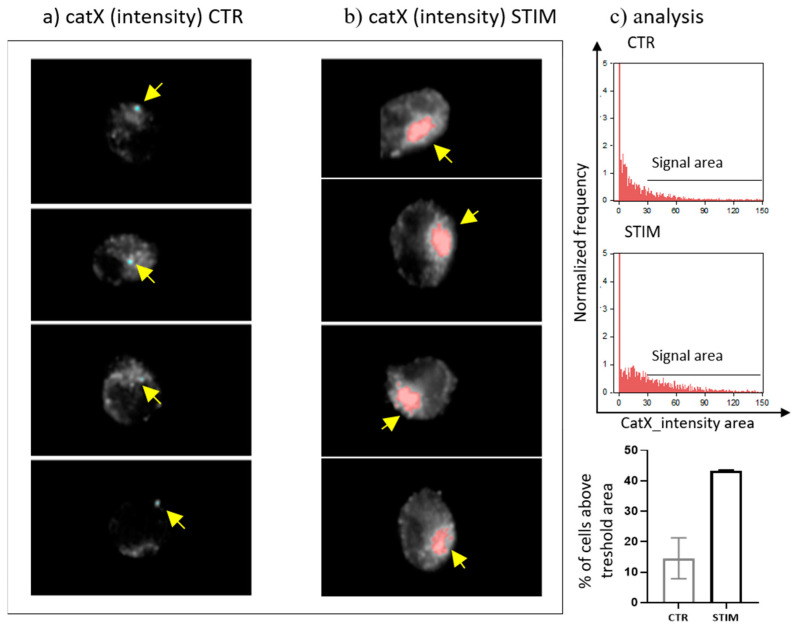
The localization of cathepsin X in NK-92 cells after PMA/IONO activation. NK-92 cells, (**a**) unstimulated (CTR) or (**b**) PMA/IONO stimulated (STIM), were stained for the imaging of intracellular cathepsin X expression by Amnis. (**c**) Post-imaging analysis in IDEAS software was used to quantify the cell area of high cathepsin X intensity. The images are representative of two independent experiments.

Since transcriptional downregulation was not the reason for the lower cathepsin X activity in activated NK-92 cells, we tested the NK-92 cell-conditioned media for the presence of cathepsin X. Cathepsin X was found only in the cell culture media of PMA/IONO-activated NK-92 cells (Figure 2a). Moreover, the majority of cathepsin X was secreted within 3 h of NK cell activation (Figure 2b), leading us to suspect that cathepsin X secretion is coupled to the cytotoxic granule exocytosis during rapid degranulation of NK cells. Indeed, analysis of cathepsin X localization in non-activated (Figure 3a, control; CTR) vs. activated NK-92 (Figure 3b, stimulated cells; STIM) cells revealed that the discrete areas of high cathepsin X intensity were larger in STIM than in CTR cells, thus indicating re-localization of cathepsin X after NK-92 cell activation. As shown, high cathepsin X intensity is locally distributed in areas as indicated by yellow arrows on the images of CTR and STIM cells on Figure 3. Of note, the mean fluorescence intensity was comparable between the two groups.

### 2.3. Cathepsin X Localization during NK-92 Cell Degranulation

**Figure 4:**
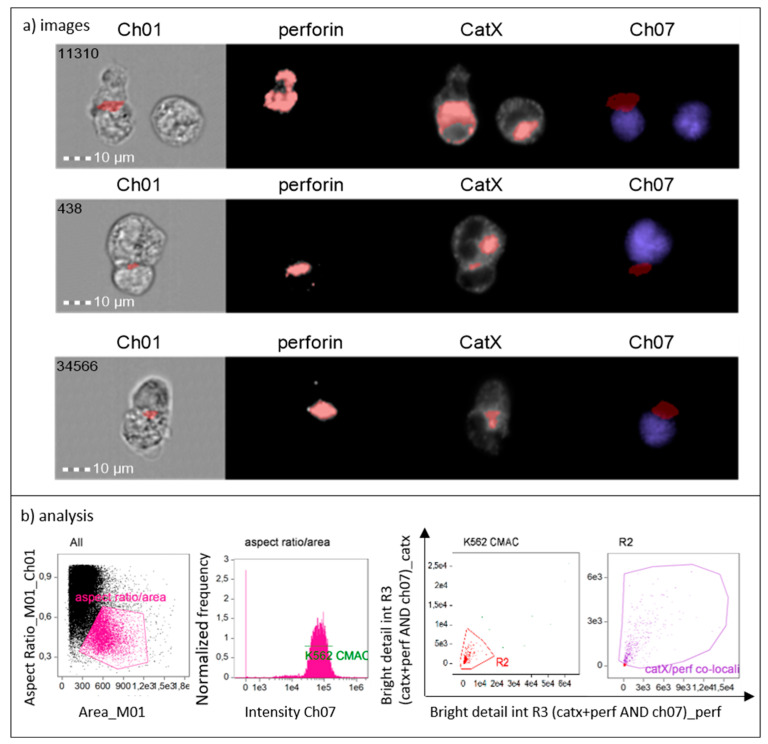
Cathepsin X is released to target cells together with perforin from NK-92 cells during cytolytic attack (**a**). Gating strategy used to isolate the positive events (NK-92 immunoconjugates) and to determine proportion of cells in which cathepsin X co-localizes with perforin (**b**).

To show that cathepsin X is preferentially concentrated in cytotoxic granules of NK-92 cells during lytic attack on targets, we incubated NK-92 cells with CMAC-labelled K-562 cells. After fixation, the cells were stained for perforin and cathepsin X. The immunoconjugates were selected based on the concomitant expression of perforin (NK-92-specific) and CMAC dye (K-562). Using IDEAS software, we created a combined mask out of individual cathepsin X and perforin masks and a CMAC dilated mask (Figure 4, Ch07 area in red). In this way, we isolated the specific area of cell conjugates, where perforin entered the target cell, for the assessment of co-localization of cathepsin X and perforin with bright detail similarity R3 feature. Colocalization of cathepsin X with perforin at the interface of the two cells was detected in ~50% of NK-92/K-562 conjugates (Figure 4, area in red).

### 2.4. Cathepsin X Vesicular Distribution and Co-Localization with LFA-1

**Figure 5:**
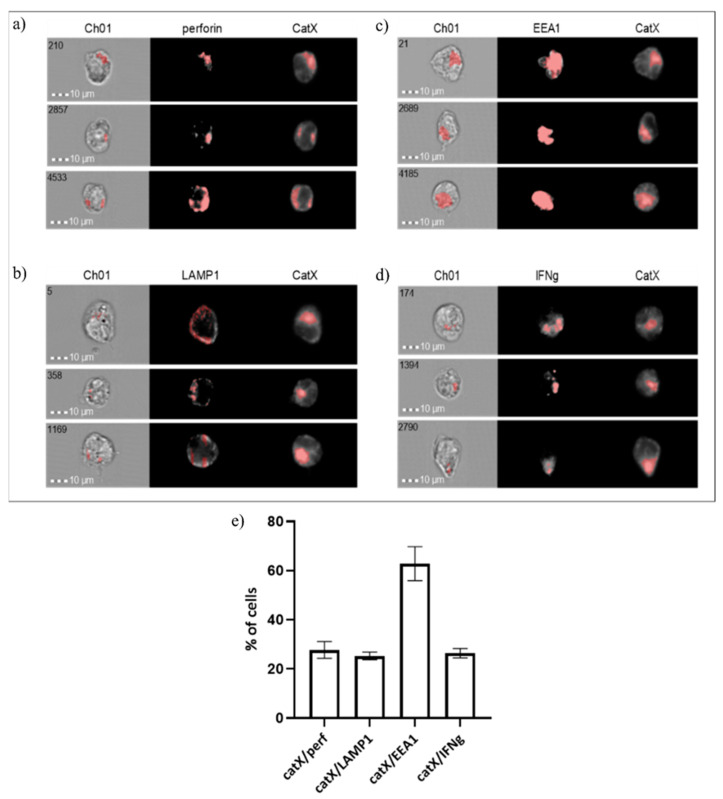
Co-localization of cathepsin X with cytotoxic granule marker perforin (**a**), lysosomal marker LAMP1 (**b**), endosomal marker EEA1 (**c**) and cytokine IFNγ (**d**) in NK-92 cells. The images are representative of the two independent experiments. (**e**) % of cells with cathepsin X co-localization.

Next, we assessed the co-localization of cathepsin X with early endosomal antigen 1 (EEA1), lysosomal-associated membrane protein 1 (LAMP1), perforin and interferon gamma (IFN-γ) in NK-92 cells (Figure 5). As expected, the proportion of cells where cathepsin X co-localized with perforin was similar to those where cathepsin X co-localized with LAMP1 (~20%). However, in the majority of cells (~60%) cathepsin X co-localized with the early endosomal marker EEA1. About 20% of cells also displayed the IFN-γ/cathepsin X colocalization.

**Figure 6:**
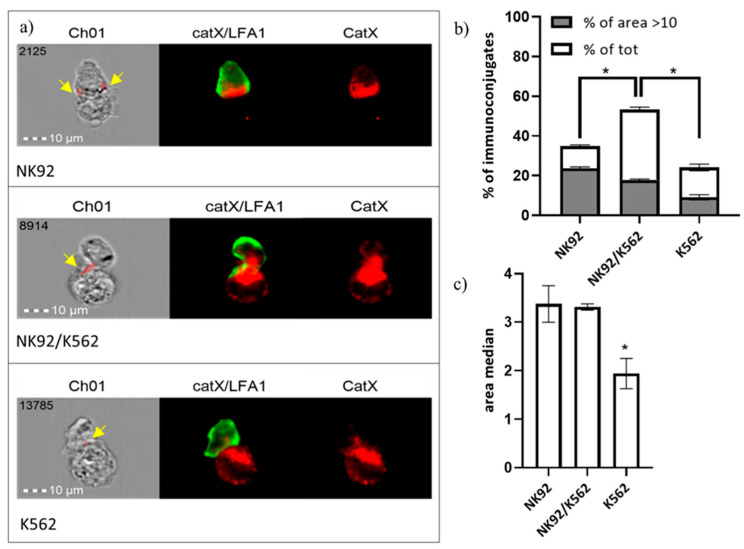
Categorization of NK-92/K-562 immunoconjugates, based on predominant cathepsin X localization. (**a**). Furthermore, the degree of cathepsin X and β2 co-localization was compared between these three groups. by comparing the size of co-localization area (**b**) and median area value (**c**). * *p* < 0.05.

In addition, we performed immunocytochemical staining of cathepsin X and integrin receptor β2 chain in NK-92/K-562 immunoconjugates. Post-imaging analysis revealed that immunoconjugates could be divided into three categories, based on predominant cathepsin X distribution (Figure 6a): “NK-92-high”, “NK-92/K-562 combined” or “K-562-high”. These categories presumably correspond to different degranulation stages of NK-92 cells. First, NK-92 cells engage their targets, and polarize cathepsin X towards interface. In the second stage, cathepsin X is being transferred to the target cells, leading to cathepsin X depletion from NK-92 cells after degranulation. For each of these categories, we determined the area of immunoconjugates, where cathepsin X signal co-localized with β2 chain (marked with yellow arrows on Figure 6a) and plotted the size distribution of co-localization area, in order to calculate proportion of cells with significant catX/β2 chain co-localization area (arbitrary set at >10; Figure 6a) and median area value (Figure 6b). Co-localization of cathepsin X and β2 chain was observed in a large proportion (53%) of immunoconjugates of the second category (NK-92/K-562) and, to a significantly smaller degree, in the first and third categories, with 34% and 24% of immunoconjugates, respectively. On the other hand, the proportion of immunoconjugates with co-localization area between cathepsin X and β2 chain >10 was highest at the first stage of degranulation (in cathepsin X enriched NK-92 cells), and gradually decreased after cathepsin X was redistributed to the target cells (Figure 6a). The overall median co-localization area value was two-fold lower for the cathepsin X-enriched K-562 category in comparison to the other two (Figure 6b). It is evident from our previous measurements (Figure 5) that not all cathepsin X co-localizes with perforin in LAMP-1 positive granules. Here, we demonstrate that cathepsin X co-localizes also with integrin β2 chain. However, their interaction depends on the stage of target cell killing and is significantly reduced after the majority of cathepsin X has been re-distributed to target cells.

Previous studies showed the possibility that secreted cathepsin X mediates biologic effects in the bystander cells [40]. To test this option, K-562 target cells were pre-incubated with one of the following: the fresh medium, the conditioned medium, that was obtained from non-activated or activated NK-92 cells, and the medium containing recombinant human cathepsin X, all of which contained either DMSO or Z9. After 24 h, the viability and proliferation of K-562 cells was measured (Supplementary Figure S2a,b). Apart from the slight increase in K-562 proliferation in the presence of the NK-92-conditioned medium, there was no difference between DMSO or Z9-treated samples. In a separate set of experiments, fresh NK-92 cells were added to the pre-conditioned K-562 cells, however, no difference in cytotoxicity was observed regardless of K-562 pre-treatment (Supplementary Figure S2c). Therefore, cathepsin X does not change the NK cell cytotoxicity or viability and proliferation of target cell and the exact role of cathepsin X accumulation and its subsequent secretion from cytotoxic granules requires further investigations.

### 2.5. Cathepsin X Inhibition Does Not Perturb Formation of Stable Immunoconjugates

**Figure 7:**
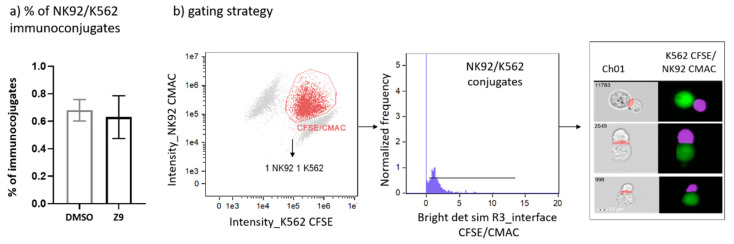
Determination of the proportion of NK-92/K-562 immunoconjugates. Comparison of NK-92/K-562 immunoconjugate frequency between DMSO and Z9-treated samples (**a**). Gating strategy used for determination of the proportion of immunoconjugates formed between NK-92 and K-562 cells (**b**). The figures are representative of two independent experiments.

Cathepsin X was shown to interfere with LFA-1 signalling pathway and to alter its adhesion to the ligand intercellular adhesion molecule 1 (ICAM-1). By disturbing LFA-1 affinity, cathepsin X could hamper interactions of NK cells with cancer cells. Therefore, we first asked whether cathepsin X contributes to the formation of stable immunoconjugates between IL-2 dependent NK cell line NK-92 and erythroleukemia line K-562. Cathepsin X-specific reversible inhibitor Z9, developed by our group [41], was used to block cathepsin X activity in NK-92 cells. To prevent excessive cytolysis, which would impede quantification of immunoconjugates, K-562 cells were only briefly brought in contact with NK-92 cells. The proportion of captured immunoconjugates formed between NK92 and K-562 is relatively low, thus we imaged at least 100,000 cells in each experiment. The proportions of such conjugates formed between DMSO-treated and Z9-treated NK-92 cells with K-562 cells were 0.68% ± 0.11% and 0.63% ± 0.22%, respectively, as depicted in Figure 7.

**Figure 8:**
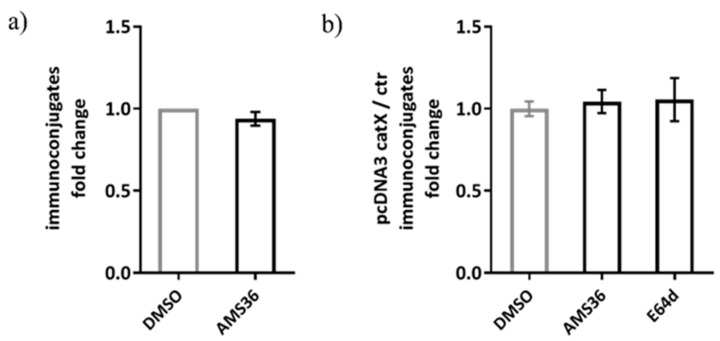
The relative comparison of SEE-specific immunoconjugate formation between DMSO or AMS36 treated-Jurkat cells with Raji cells. The proportion of immunoconjugates was determined by performing flow cytometry and gating the doublets positive for CMAC-labelled Jurkat and CMTMR-labelled Raji cells in FlowJo software. The relative comparison between vehicle and inhibitor-treated cells was made for wild type Jurkat cells (**a**) and for cathepsin X-overexpressing Jurkat cells (**b**). The results represent the mean ± SD of the three independent experiments.

Previous work demonstrated the association between cathepsin X and LFA-1 in Jurkat cells. Therefore, we tested our hypothesis on alternative model, consisting of Jurkat non-cytotoxic T cell line interacting with SEE-coated Raji B cell line, to simulate immunological synapse during antigen presentation instead of target cell killing. The activation of Jurkat cells upon contact with the SEE-coated Raji cells was confirmed by measuring CD69 expression and the appropriate controls were used to confirm that there was negligible unspecific binding between Jurkat and Raji cells (Supplementary Figure S3a). First, we tested wild-type Jurkat cells, and found no significant difference between DMSO and cathepsin X inhibitor pre-treated Jurkat cells in the formation of stable conjugates with Raji cells (Figure 8a). Here, the same irreversible cathepsin X inhibitor AMS36 was used as in the study by Jevnikar et al., where the addition of AMS36 reduced association of talin with β_2_ integrin chain in Jurkat cells and prevented conversion of active LFA-1 to high affinity conformation [26]. Next, we transfected Jurkat cells with pcDNA3/cathepsin X, since it was previously shown that cathepsin X overexpression enhances migration and elongated tube formation in Jurkat cells and that both processes could be inhibited by AMS36 [29,30]. Cathepsin X upregulation in Jurkat cells after transfection was demonstrated by Western blot (Supplementary Figure S3b). However, cathepsin X-transfected cells did not differ from mock-transfected cells in their ability to form stable conjugates, neither after addition of AMS36 nor of broad-spectrum cysteine cathepsin inhibitor E64-d (Figure 8b), thus excluding the involvement of cathepsin X, or any other cysteine cathepsin, in maintaining immunoconjugate stability. Of note, we also incubated primary CD4^+^ T cells with SEE-loaded Raji cells for the imaging of immunoconjugates on Amnis, and, again, no difference was detected between DMSO- and AMS36-treated primary T cells (data not shown).

### 2.6. Cathepsin X Inhibition Does Not Perturb NK-92 Cell Lytic Activity

**Figure 9:**
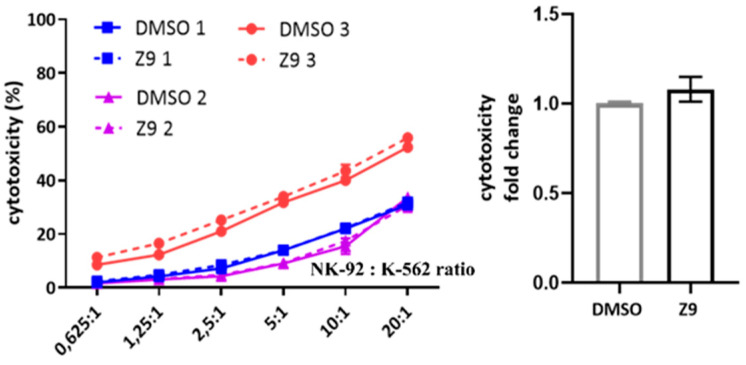
The effect of cathepsin X inhibition on NK-92 cell cytotoxicity. NK-92 cells were pre-incubated with DMSO or Z9 and brought into contact with CFSE-labelled K-562 target cells to initiate killing. Afterwards, the cells were collected, stained with viability dye 7-AAD and analysed by flow cytometry. The NK-92 cell cytotoxicity was determined by calculating the fraction of CFSE+/7-AAD+ events relative to all CFSE+ target cells, which was plotted against different effector to target cell ratios, to obtain the killing curves (**left**). From killing curves of three independent experiments, the lytic activity was calculated and normalized to DMSO control for comparison of relative change in cytotoxic activity of DMSO- vs Z9-treated NK-92 cells (**right**). The results represent the mean ± SD of the three independent experiments.

Finally, we considered the possibility that cathepsin X could be involved in NK cell cytotoxicity. NK-92 cells were treated with cathepsin X inhibitor Z9 and their cytotoxicity was evaluated in a killing assay against K-562 target cell line. The inhibition of cathepsin X by Z9 did not significantly impact NK-92 cytotoxicity (Figure 9) and neither did inhibition with AMS36 or E64-d (Supplementary Figure S4a). Of note, we demonstrated the reduction of cathepsin X activity in whole cell lysates after the cells were incubated with AMS36 for 2 h or 24 h (~30%; Supplementary Figure S4b). Z9 is reversible inhibitor and thus reduction of cathepsin X activity cannot be observed in kinetic assay. Nevertheless, it was previously shown that Z9 could permeate the cells [41].

## 3. Discussion

The integrin receptor LFA-1 mediates cell-cell interaction and firm adhesion of leukocytes, including the initiation of the conjugate formation between NK cell and its target. In addition, LFA-1 is required for the polarization of the lytic granules towards target cells, thus affecting NK cell cytotoxicity [42,43]. LFA-1 adhesiveness is regulated by the integration of various signals that are transduced from the “outside–in” as well as from the “inside–out”. LFA-1 is a heterodimer of α_L_ subunit, which defines ligand-binding specificity, and β_2_ subunit. The β_2_ chain is particularly important for LFA-1 connection to the cytoskeleton and for transmission of conformational changes that fine tune the binding affinity for the ligand ICAM-1, ranging from the bent, low affinity, to the extended, closed and open conformation with intermediate and high affinity, respectively [44,45]. It was shown that cathepsin X sequentially cleaves four C-terminal amino acids from β_2_ cytoplasmic tail, thus modulating its affinity for the binding of adaptor proteins [26,46]. Our goal was to explore the impact of cathepsin X activity on LFA-1 mediated functions of NK cell immune synapse.

Cysteine cathepsins are considered as important targets in anti-cancer therapy, since their increased activity drives pathologic processes that are associated with tumour growth, invasion and immune cell dysfunction. Small molecule synthetic inhibitors of the more studied cathepsins B, S and K have already entered clinical trials [11]. For cathepsin X, the recently developed triazole-based reversible inhibitor showed promising anti-tumour activity in vitro [41] and in vivo (Mitrović et al., publication in preparation) and could be used in addition to irreversible inhibitors, like AMS36. However, besides their impact on tumour progression, several studies have highlighted possible impact of cysteine peptidase inhibitors on different immune cell subsets. For example, the inhibition of cathepsins influenced the development of osteoclasts from immunosuppressive myeloid-derived suppressor cells, which could contribute to bone metastases [47]. In contrast, the inhibition of cathepsin S was shown to convert regulatory T lymphocytes from immunosuppressive to immunostimulatory cells [48] and inhibition of cathepsin L enhanced cytotoxicity of CD8^+^ T cells in the co-culture model of tumour cells and myeloid-derived suppressor cells [25]. With this in mind, we investigated whether the inhibition of cathepsin X activity in cytotoxic cells influences LFA-1 affinity modulation, and thus effects formation of stable immunological synapse and cytotoxic activity of NK-92 cells. Importantly, our data showed that cathepsin X inhibition has no impact on the formation of stable immunoconjugates between NK-92 cells and target cells, nor did cathepsin X inhibition change NK-92 cell cytotoxicity against the target cells. These findings were corroborated by imaging flow cytometry analysis of cathepsin X subcellular localization in resting and activated NK-92 cells.

We demonstrated that the cathepsin X inhibitor AMS36 reduced cathepsin X activity in NK-92 cells by~30%. In our previous study, the 25% reduction of cathepsin X levels in CD4^+^ T cells was sufficient to reduce LFA-1 affinity by 26% resulting in lower CD4^+^ T cell spreading on SDF-1/ICAM-1 coated surface [26]. However, it was demonstrated that LFA-1 activation and affinity regulation is dependent on the type of the activating stimulus and differs between chemokine or T-cell-receptor-stimulated cells [49]. Furthermore, the interaction of cytotoxic cells with target cells is much more complex than the interaction with ligand-coated surfaces. Cytotoxic cells receive numerous activating and inhibitory signals through a wide variety of receptors, which act in synergy to enhance individual signalling pathways [42]. It is possible that the co-stimulation through other receptors enhances LFA-1 affinity, in spite of cathepsin X inhibition. In addition, the interactions between integrin receptors and cytoskeleton are not regulated only through proteolytic cleavage but could be modified by phosphorylation of tyrosine/serine residues in the cytoplasmic domains of α and β subunits. Indeed, phosphorylation of Ser745 and Thr758 was shown to be important for affinity control of LFA-1 in T-cell adhesion to ICAM-1 [50,51,52]. The involvement of other adhesion receptors might also partially compensate for the loss of LFA-1 adhesiveness [53,54].

On the other hand, our data highlight another important function cathepsin X may play in NK cells, i.e., the involvement in degranulation and cell cytotoxicity. We demonstrated that cathepsin X is secreted from activated NK-92 cells during cell degranulation. We confirmed the presence of cathepsin X in cytotoxic granules of NK-92 cells by co-localization of cathepsin X with perforin and showed their entry to the target cell by using imaging flow cytometry. While NK-92 cells transfer majority of cathepsin X to target cells, only a fraction of cathepsin X is secreted associated with perforin. Large portion of cathepsin X is present in EEA1^+^ and IFNγ^+^ vesicles as well. In human primary NK cells IFNγ was shown to be trafficked and secreted independently of perforin [55], which could explain alternative route for cathepsin X entry into target cells. Even though the addition of exogenous cathepsin X didn’t have detrimental effects on target cell fitness, we cannot exclude alternative roles of secreted cathepsin X.

In the previous studies, partial loss of cathepsin X activity due to inhibition or siRNA silencing had significant impact on LFA-1 mediated signalling and influenced integrin-dependent homotypic aggregation, migration, and cytoskeletal rearrangement of CD4^+^ T cells [30]. Additionally, cathepsin X was shown to translocate to the peri-membranous region of activated CD4^+^ T cells to co-localize with LFA-1 [30] and activate it. Here, we show that cathepsin X transiently co-localizes with integrin β_2_ chain during degranulation of NK92 cells and is preferentially redistributed to target cells. Neither of cathepsin X inhibitors used in this study affected LFA-1 dependent synapse stability and target cell lysis by NK-92 cells, which are both sensitive to alterations in LFA-1 affinity. Altogether, our data suggest that cathepsin X inhibition, potentially used in anticancer therapy, is not detrimental to the NK cell cytolytic activity and would not impair immune synapse formation, nor the cytotoxic activity of NK cells. That said, additional studies to identify role of cathepsin X in cytotoxic granules could be of further interest.

## 4. Materials and Methods

### 4.1. Cell Lines

The NK-92, K-562, Jurkat and Raji cell lines were all obtained from ATCC (Manassas, VA, USA) and were maintained in complete advanced RPMI 1640 medium (Gibco, Waltham, MA, USA). Medium was supplemented with either 12.5% foetal bovine serum (FBS) and 12.5% horse serum (Gibco, Waltham, MA, USA) for NK-92 or 10% FBS for K-562, Jurkat and Raji cells. Complete media also contained 2mM L-glutamine and 100 U/mL penicillin, 0.1 mg/mL streptomycin (Sigma-Aldrich, St. Louis, MO, USA). To sustain proliferation of interleukin (IL)-2-dependent NK-92 cells, 200 U/mL of recombinant human IL-2 (Bachem, Bubendorf, Switzerland) was added periodically to the culture. In order to activate maximal lytic activity, NK-92 cells were incubated overnight with 1000 U/mL IL-2 before cytotoxicity experiments.

### 4.2. Cell Lysate Preparation for Measuring Cathepsin X Activity, mRNA Expression and Protein Levels

Cells were stimulated with 1000 U/mL IL-2, 50 ng/mL PMA (Sigma-Aldrich, St. Louis, MO, USA) and/or 0.5 µM ionomycin (Sigma-Aldrich, St. Louis, MO, USA) or DMSO (Sigma-Aldrich, St. Louis, MO, USA) vehicle. After indicated time points, cells were harvested, washed with PBS and lysed in RIPA buffer [150 mM NaCl, 50 mM Tris, 1% Nonidet P-40, 0.5% Na-deoxycholate, 0.1% Na-dodecyl sulfate (SDS), 0.004% Na-azide, pH 8.0] for protein expression analysis by Western blot or in buffer for measuring cathepsin X activity [50 mM Na-acetate, 1 mM EDTA, 0.1 M NaCl, 0.25% Triton X-100, pH 5.5]. Whole cell lysates were stored at −80 °C, freeze thawed and centrifuged at 16,000× *g* for 15 min at 4 °C. Protein concentration in collected supernatants was determined with a DC-Protein Assay kit (Bio-Rad Laboratories, Hercules, CA, USA). For RNA isolation, cells were pelleted and stored in 1 mL RiboZol (VWR Chemicals, Radnor, PA, USA) at −80 °C until further processing.

### 4.3. Kinetic Assay

For evaluating cathepsin X activity, cell lysates were diluted to 0.125 mg/mL protein concentration in 100 mM acetate buffer pH 5.5 [15 mM acetic acid, 84.8 mM Na-acetate, 0.1% PEG 8000] with 5 mM L-cysteine and 1.5 mM EDTA. 50 uL of lysates were pipetted in duplicates to the wells of the black microtiter plate (Nunclon Delta Surface; Thermo Fisher Scientific, Waltham, MA, USA) and incubated at 37 °C for 10 min. Immediately, 50 µL of fluorogenic substrate Abz-FEK(Dnp)-OH [56] at 5.9 µM final concentration was added and its degradation was monitored continuously at 320 ± 5 nm excitation and 420 ± 5 nm emission with a spectrophotometer Tecan Safire (Tecan, Männedorf, Switzerland). Kinetic measurements were analysed by Magellan™ [57] software and data, expressed in RFU/sec, were normalised to the control sample at 0 h.

### 4.4. Western Blotting

Fifteen micrograms of proteins from whole cell lysates were denatured by addition of SDS-page buffer, heated for 10 min and resolved by SDS-PAGE on 12% Tris-glycine gels and then transferred to nitrocellulose membrane using iBlot (Thermo Fisher Scientific, Waltham, MA, USA). The membranes were blocked in 5% non-fat dried milk in Tris buffered saline with 0.05% Tween-20 and probed with goat anti-cathepsin X (1/1000; AF934; R and D Systems, Minneapolis, MN, USA) and rabbit anti-β-actin (1/3000; A2066; Sigma-Aldrich, St. Louis, MO, USA) antibodies. After washing, secondary HRP-conjugated anti-goat (1/3000; sc-2354; Santa Cruz Biotechnology, Dallas, TX, USA) and anti-rabbit (1/5000; 111-035-045; Jackson ImmunoResearch, West Grove, PA, USA) antibodies were added and protein bands were visualized with Super Signal West Dura Extended Duration Substrate (Thermo Fisher Scientific, Waltham, MA, USA) and recorded with G:Box imager (Syngene, Bengaluru, Karnataka, India). The band intensities were quantified using the Gene Tools software [58] and normalised to control sample at 0 h.

### 4.5. RNA Isolation and Quantitative Real-Time PCR Analysis

Total RNA was extracted from NK-92 cells with phenol/chloroform, followed by cleaning step with 5Prime Phase Lock Gel (Quantabio, Beverly, MA, US) and ethanol precipitation. One microgram of total RNA was reverse transcribed using oligo(dT)_18_ primers with RevertAid First Strand cDNA Synthesis Kit (Thermo Fisher Scientific, Waltham, MA, USA) according to the manufacturer’s instructions. Two-step qPCR reactions were performed in triplicates using Maxima SYBR Green/ROX qPCR Master Mix 2X (Thermo Fisher Scientific, Waltham, MA, USA) by adding 1.0 ng cDNA at 10 µL volume and were run with LightCycler 480 (Roche Diagnostics, Basel, Switzerland) at following conditions: 10 min at 95 °C, followed by 40 cycles at 95 °C for 15 sec and at 60 °C for 1 min. Primers were designed with NCBI Primer-BLAST (accessed on 19 February 2020)-Table 1, validated by melting curve analysis and reaction efficiencies were confirmed to be within 90% to 110%. GAPDH was selected as the optimal reference gene by NormFinder [59]. Data were calculated according to the guidelines by Taylor et al. [60].

### 4.6. Collection of Concentrated Cell CULTURE Media

For detection of cathepsin X secretion in cell culture media, NK-92 cells were treated in serum-free medium (SFM) and collected after the indicated time points. Supernatants were removed following 10-min centrifugation at 4 °C, 2500 rpm and concentrated by using Amicon’s Centricon Centrifugal Filter Units (Merck Millipore, Burlington, MA, USA). Equal aliquots of supernatants were prepared for Western blotting, as described above.

### 4.7. Imaging Flow Cytometry for Co-Localization Analysis

For the inspection of subcellular localization of cathepsin X in NK-92, cells were fixed with 4% paraformaldehyde (Sigma-Aldrich, St. Louis, MO, USA) for 20 min at room temperature, washed with PBS buffer containing 1% bovine serum albumin (BSA; Sigma-Aldrich, St. Louis, MO, USA) and permeabilized for 5 min with 0.5% Tween-20 (Sigma-Aldrich, St. Louis, MO, USA) in PBS. Cells were washed and a blocking solution of 4% donkey serum (Sigma-Aldrich, St. Louis, MO, USA) in PBS was added for 30 min before proceeding to immunolabeling with the following primary antibodies: rabbit anti-LAMP1 (1/200; SAB3500285; Sigma-Aldrich, St. Louis, MO, USA), rabbit anti-EEA1 (1/400; C45B10; Cell Signalling Technologies, Danvers, MA, USA), mouse anti-perforin (1/400; dG9; BioLegend, San Diego, CA, USA), mouse anti-interferon γ (IFN-γ) (1/400; 4S.B3; BioLegend, San Diego, CA, USA), mouse anti-CD18 (1/100; TS1/18; Invitrogen, Waltham, MA, USA) and goat-anti cathepsin X (1/100; AF934; R and D Systems, Minneapolis, MN, USA). After 45-min incubation at 4 °C, the primary antibodies were washed and substituted with solutions containing donkey -anti-goat Alexa Fluor 647, -anti-mouse or -anti-rabbit Alexa Fluor 488 secondary antibodies (1/1000; Invitrogen, Waltham, MA, USA) for 30 min at room temperature. Finally, the samples were resuspended in PBS and 10.000 images of NK-92 cells were captured on Amnis ImageStream Mk II (Luminex, Austin, TX, USA) imaging flow cytometer. Data were analysed with the IDEAS software [61].

Colocalization of cathepsin X with perforin NK-92/K-562 immunoconjugate was also imaged on Amnis. For image analysis, CMAC target cell mask was combined with perforin and cathepsin X mask to define the area at the interface of NK-92/K-562 immunoconjugate, where perforin enters the target cells (Ch07, red mask). The mask was then used to gate the immunoconjugates, in which cathepsin X colocalized with perforin (Ch01, red mask). Similarly, the intensity masks were set for each fluorescence channel and the bright detail similarity feature was used to define the proportion of cells, where cathepsin X colocalized with the each of the following markers: EEA1/LAMP1/perforin/IFNγ.

### 4.8. Immunoconjugate Stability Assay

Immunoconjugate stability of NK-92/K-562 and Jurkat/Raji cells was assessed according to the modified procedure, reported by Na et al. [62] Raji cells were washed with serum-free medium and incubated with 2 µg/mL staphylococcal enterotoxin E (SEE; Toxin Technologies, Sarasota, FL, USA) for 45 min. During last 15 min CellTracker Orange (CMTMR; Invitrogen, Waltham, MA, USA) was added to final concentration of 5 µM, then labelling reaction was terminated by addition of complete medium. Jurkat cells were pre-incubated either with DMSO (vehicle control; Sigma-Aldrich, St. Louis, MO, USA) or cathepsin X inhibitor AMS36 (10 µM) for 2 h prior to labelling with CellTracker Blue (CMAC; Invitrogen, Waltham, MA, USA) as described above for Raji cells. 2 × 10^5^/200 uL of each cell type was aliquoted in flow tubes and samples were briefly spun at 200× *g* for 1 min to allow immunoconjugate formation between SEE-loaded Raji cells and antigen-sensing Jurkat cells. After 15-min incubation at 37 °C, 5% CO_2_, cells were washed with PBS, mixed well in order to detach unspecific binding between cells, and analysed by flow cytometry (Attune NxT; Thermo Fisher Scientific, Waltham, MA, USA). Proportion of immunoconjugates (CMTMR^+^CMAC^+^ events) was determined with FlowJo software [63]. In the case of NK-92/K-562 conjugates, cells were first labelled with CMAC and CFSE, respectively. NK-92 cells were pre-incubated either with DMSO or cathepsin X inhibitor Z9 (10 µM) for 2 h. Next, they were joined with K-562 target cells in 1:1 ratio, briefly centrifuged, and allowed to interact for 5 min before fixation with 4% paraformaldehyde. The cells were then washed in PBS and 100.000 events were recorded by imaging flow cytometry (Amnis ImageStream Mk II; Luminex, Ashland, OR, USA). Immunoconjugates between NK-92 and K-562 cells were visualized and quantified with IDEAS analysis software [61].

After imaging, the proportion of immunoconjugates was evaluated by first gating the doublets that contain one of each CFSE+ K-562 and CMAC+ NK-92 cell, and then by confirming the close proximity of the two interacting cells. This was done by identifying the intersection area, consisting of CFSE and CMAC masks, which were dilated by three pixels. Next, the bright detail similarity R3 feature was used, in order to compare the small bright image detail of the two images (i.e., CFSE and CMAC) and to determine the degree of co-localization of these two signals in the pre-defined intersection area.

### 4.9. Transient Transfection

The pcDNA3/cathepsin X plasmid was prepared as described in Jevnikar et al. [30], transformed and propagated in *E. coli* and purified using GenElute Miniprep Kit (Sigma-Aldrich, St. Louis, MO, USA). Jurkat cells were seeded in 24-well plates at 2 × 10^5^ cells/500 µL medium without antibiotic. Lipofection was performed the next day using Lipofectamine 2000 (Invitrogen, Waltham, MA, USA) according to the manufacturer´s instructions. Briefly, 18 µg of DNA plasmid in 300 uL medium without antibiotic was mixed with an equal amount of medium containing 12 uL Lipofectamine and incubated for 20 min at room temperature. Transfection medium was added to the cells dropwise and cells were placed on an orbital shaker for 15 min. Six hours post transfection the transfection medium was replaced with complete medium. After 24 h cells were aliquoted and cell lysates were collected for evaluation of transfection efficiency by Western blotting or they were used in immunoconjugate stability assay.

### 4.10. Cytotoxicity Assay

Cytotoxicity of NK-92 cells was evaluated by measuring lysis of target cells K-562. Briefly, K-562 were labelled with 0.2 µM CFSE (Invitrogen, Waltham, MA, USA) for 10 min and were added to effector cells in 96-well U-bottom plates, spun for 1 min at 200× *g* and incubated for 2.5 h. Afterwards, the samples were collected, stained on ice with 7-AAD (Sigma-Aldrich, St. Louis, MO, USA) for 10 min and analysed by flow cytometry. Proportion of CFSE^+^/7-AAD^+^ events was determined for each effector cell/target cell ratio (E:T) and lytic activity (LU) was determined as described in [64].

### 4.11. Statistics

Statistical calculations were performed in GraphPad Prism [65] using one-way ANOVA or Student’s *t*-test.

## Figures and Tables

**Table 1:** Primer pair sequences for qPCR.

Gene	Primer Pair	Sequence (5′→ 3′)
CtsZ	F R	TGAACCATGGGGCGAGAGAG AGTGCTCCTCGATGGCAAGG
GAPDH	F R	TGCACCACCAACTGCTTAGC TGGCATGGACTGTGGTCATG

## Supplementary Materials

The following are available online at https://www.mdpi.com/article/10.3390/ijms222413495/s1.

## Abbreviations

**NK**—natural killer; **7-AAD**—7-aminoactinomycin D; **CFSE**—carboxyfluorescein succinimidyl ester; **FBS**—fetal bovine serum; **CMAC**—7-amino-4-chloromethylcoumarin; **CMTMR**—5-(and-6)-(((4-chloromethyl)benzoyl)amino)tetramethylrhodamine; **SEE**—staphylococcal enterotoxin E; **IL**—interleukin; **LFA-1**—lymphocyte function-associated antigen 1; **LAMP-1**—lysosomal-associated membrane protein 1; **EEA1**—early endosomal antigen 1; **IFNγ**—interferon gamma; **ICAM-1**—intercellular adhesion molecule 1.
