# Supplementary material for: Cathepsin X Activity Does Not Affect NK-Target Cell Synapse but Is Rather Distributed to Cytotoxic Granules
Source: Int J Mol Sci. 2021 Dec 16;22(24):13495. doi: 10.3390/ijms222413495 (PMC8707301; doi:10.3390/ijms222413495)
Supplement: Supplementary file 1 [file ijms-22-13495-s001.zip › ijms-1497683-supplementary.pdf]

Supplementary Figure S1

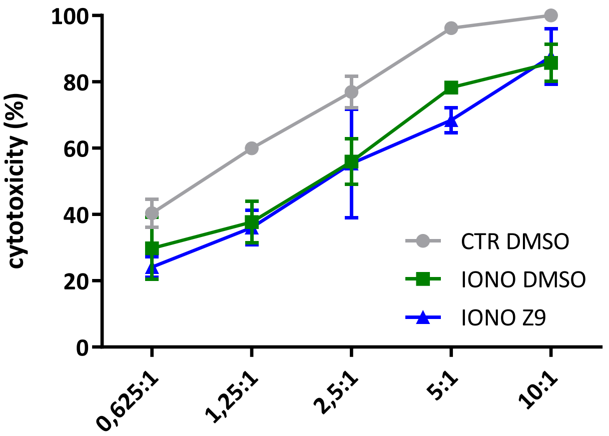

Supplementary Figure S1 Increased cathepsin X activity after ionomycin treatment of NK-92 cells does not change NK-92 cell cytotoxicity. NK-92 cells were treated overnight with IONO or vehicle (DMSO). 24 h before the cytotoxicity assay, DMSO or Z9 were added to IONO treated cells. The cytotoxicity of NK-92 cells was significantly reduced with IONO treatment, however addition of Z9 did not further influence NK-92 cell cytotoxicity. The figure represents two independent experiments.

Supplementary Figure S2

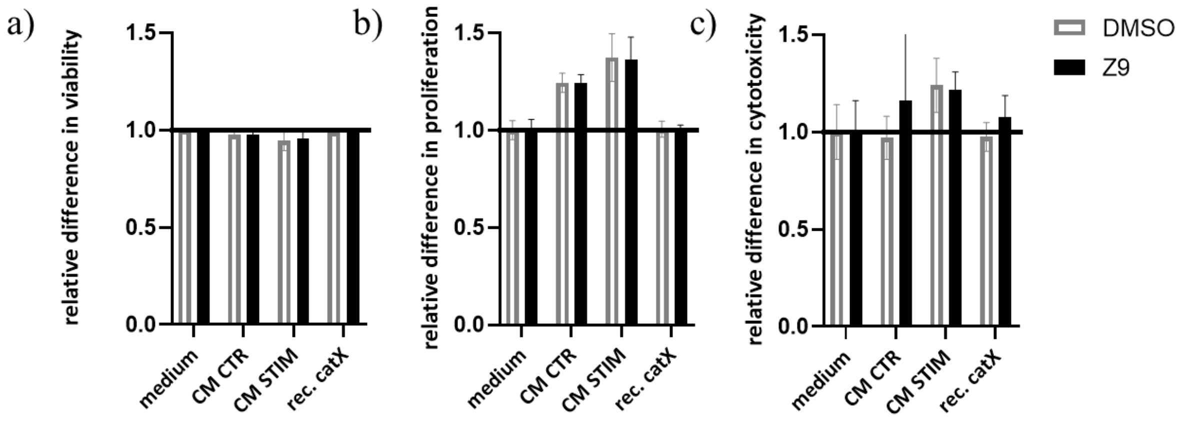

Supplementary Figure S2 Cathepsin X, that is secreted by NK-92 cells, does not influence K-562 viability (a), proliferation (b) nor their susceptibility to the cell lysis by NK-92 (c). NK-92 (control and PMA/IONO stimulated) were cultured for 24 h before the collection of cell culture medium. DMSO or Z9 were added to the filtered conditioned medium from NK-92 cells, in addition to the fresh cell culture medium and cell culture medium containing 0.5 μg/mL of recombinant human cathepsin X. Equal amounts of CFSE-labelled K-562 cells were incubated for 24 h with the media so prepared, before measuring the variations in their viability, proliferation and susceptibility to the NK-92 cell lysis. The figure represents mean ± SD of two independent experiments.

Supplementary Figure S3

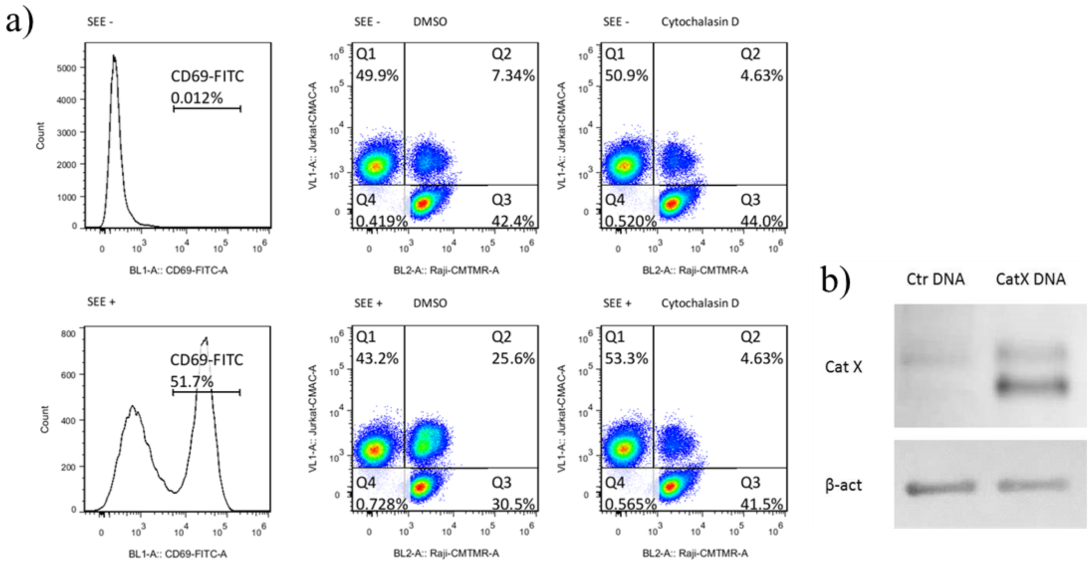

Supplementary Figure S3 Control experiments that show suitability of Jurkat/Raji immunoconjugates for studying the impact of cathepsin X inhibition. a) Jurkat cells form stable immunoconjugates with Raji cells only in the presence of SEE. Jurkat cells were incubated with Raji cells or SEE-loaded Raji cells (1:1). To confirm the SEE-specific activation of Jurkat cells, expression of CD69 was measured by flow cytometry and it was detected only in the presence of SEE (left). As a positive control, cytochalasin D was used to demonstrate disruption of immunoconjugates by this actin depolymerizing agent (right). b) Western blot analysis of cathepsin X overexpression in Jurkat cells. 24 h after transfection the levels of cathepsin X increased in pcDNA3/catX transfected Jurkat cells in comparison to the mock transfected cells (β-actin was used as loading control). The figures are representative of two independent experiments.

Supplementary Figure S4

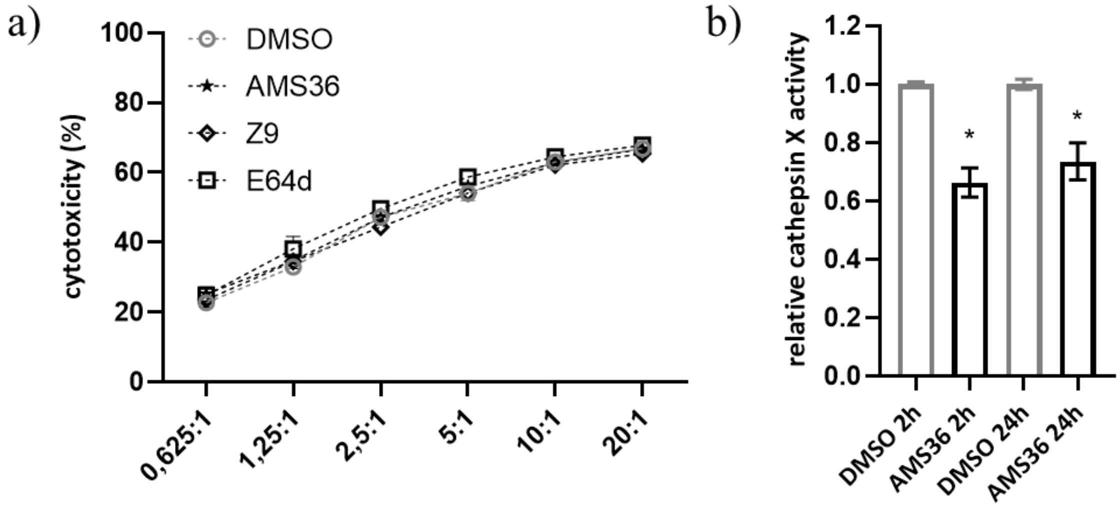

Supplementary Figure S4 The impact of selective cathepsin inhibitors and broad spectrum cathepsin inhibitor on NK-92 cell cytotoxicity. a) NK-92 cells were pre-treated with DMSO, selective cathepsin X inhibitors Z9 (10 μM) and AMS36 (10 μM) or broad-spectrum cathepsin inhibitor E64-d (10 μM) for 2h before the addition of CFSE-labelled K562 target cells. To detect target cells that were killed by NK-92 cells, 7-AAD was added and the samples were analyzed by flow cytometry. The figure is representative of multiple independent experiments. b) AMS36 reduced intracellular cathepsin X activity. To demonstrate that AMS36 blocks intracellular cathepsin X activity, the NK-92 cells were treated for 2 h and 24 h with 10 μM AMS36 before collection and resuspension in lysis buffer for measuring cathepsin X activity. Results were plotted as mean ± SD of three independent biological replicates. * $p < 0.05$.
